# Human bocavirus is detected in human placenta and aborted tissues

**DOI:** 10.1111/irv.12609

**Published:** 2018-09-30

**Authors:** Max Hansen, Michael Brockmann, Verena Schildgen, Oliver Schildgen

**Affiliations:** ^1^ Institut für Pathologie Kliniken der Stadt Köln gGmbH Klinikum der Privaten Universität Witten/Herdecke mit Sitz in Köln Cologne Germany

## Abstract

**Background:**

To date, four human bocaviruses (HBoV) have been described. The most closely related viruses (bovine and canine parvoviruses) are associated with miscarriage in their hosts. The objective of this retrospective study was to determine the frequency of HBoV DNA in miscarriage.

**Study Design:**

Tissue samples from 172 patients, in which miscarriage occurred, were included and tested with a published qPCR protocol. Positive PCRs were mutually confirmed by sequencing.

**Results:**

43 patients (25%) were positive for HBoV DNA. Of those, the majority of HBoV‐positive samples were tissues from miscarriage (placenta: 6; aborted tissue products of conception: 37 specimens). The samples were not paired; either placental or aborted tissue was available.

**Conclusions:**

The results show that, as long as no animal model is available, the role of HBoV in the occurrence of miscarriage requires additional prospective studies in order to investigate its significance and causal involvements of this pathogen.

## 1 BACKGROUND

The human bocavirus was detected in 2005,^1^ covers 4 distinct subtypes^2^, ^3^ and is associated with respiratory infections and gastroenteritis.^4^ HBoV is distributed worldwide, with an antibody sero‐prevalence up to 95% in children ≥5 years (reviewed by^5^, ^6^), and the percentage of patients in whom the virus is able to persist is up to 50%.^7^ HBoV1‐HBoV4 along with gorilla bocavirus belong to the genus Bocaparvovirus, in which HBoV1 and HBoV3 are members of the species Primate bocaparvovirus 1, whereas HBoV2 and HBoV4 belong to the species Primate bocaparvovirus 2 (https://talk.ictvonline.org/). The most closely related viruses are veterinary pathogens infecting cattle and dogs, known as bovine and canine parvoviruses.^8^

These pathogens, besides other clinical symptoms, are responsible for abortions in their natural hosts.^9^, ^10^, ^11^ However, there are solely limited data on the role of HBoV in miscarriage and it was not detected yet,^12^, ^13^ although the human parvovirus B19, as the only other human pathogenic parvovirus, is long known to be teratogenic and cause serious malformations and abortions during pregnancy.^14^

## 2 OBJECTIVES

The objective of this study was to empirically analyze the percentage of HBoV DNA in placenta and aborted tissue products of conception of miscarriages with unknown etiology.

### 2.1 Study design

A total number of 172 patients were included. Of those, aborted tissues samples were obtained from 148 patients, placenta samples were obtained from 24 patients. Due to the retrospective design of the study, in none of the cases paired samples were available, thus solely one sample per patient was available. When the miscarriage was clinically reported, the tissues were sent to the pathology lab to be investigated by histology. The sample selection was described in our earlier study in which we screened samples from miscarriage and spontaneous abortion of unknown origin for the presence of Zikavirus RNA.^15^ Remaining samples of this earlier study were included in the present investigation.

As control group, we included 19 samples from elective interruptions of pregnancy, of those 10 placenta tissues from sibling births (ie, Caesarean section, healthy newborns) and 9 embryonic tissues which had malformations diagnosed previously by ultrasound examination. The number of included controls was limited due to the fact that the cases rarely present to our hospital.

All samples were available as FFPE specimens. DNA was extracted using the Maxwell FFPE DNA kit (Promega, Darmstadt, Germany). From all samples, qPCRs that detect and differentiate the HBoV strains 1‐4 were performed as previously described by Kantola and coworkers^3^ with the exception that no quantification was performed because no reliable ratio between copy number and amount of FFPE tissue could be determined. Control plasmids/standards were kindly provided by Maria Söderlund‐Venermo, Haartman Institute Helsinki, Finland. Positive PCRs were mutually confirmed by cloning and sequencing of the PCR product.

## 3 RESULTS

The results are summarized in Table 1. Of the 172 tissues from 172 patients, 43 (25%) were positive for at least one HBoV strain. Of those 43 samples, 6 samples were from placenta tissue, specimens from 37 patients were aborted tissue products of conception, of those 10 with histologically confirmed embryonic tissue portions. The gestational age of the aborted tissues ranged from 6 to 15 weeks (mean: 9.78 weeks, median 10 weeks). In none of the samples, any specific histological signs of inflammation were observed, and most samples were categorized as missed abortion. In none of the cases, fetal tissues were observed by histological investigation, while embryonic tissues were identified by histological analyses in 11 cases. In those cases, in which the placenta tissue was HBoV‐positive, placental abruption was observed in 4 cases 5 to 1 weeks before the predicted date of delivery. One case resulted in a stillbirth at gestation week 38 with histologically confirmed signs of fibrosis of the villi, the last case was accompanied by a gestational diabetes.

**Table 1:** Summary of the major results and cohort characteristics

	No. of patients	No of specimens	HBoV‐positive	Percentage HBoV‐positive
Placenta	24	24	6	25%
Aborted tissue products of conception	148	148	37	25%
Total	172	172	43	25%
Placenta controls	10	10	0	0%
Aborted tissue products of conception control, elective abortions	9	9	0	0%

HBoV‐1 was detected in 12 cases, HBoV‐3 in 5 cases, and the residual cases were HBoV types 2 and 4 positive. Mixed infections were observed in 3 cases, of those 2 with HBoV 1 and 3, and one with HBoV‐1 and 2/4. Positive DNA detections were mutually performed by cloning and sequencing and confirmed the PCR results.

In the control group consisting of placenta samples from otherwise healthy sibling Caesarian sections and embryonic tissues with malformations of non‐infectious origin, no samples were tested positive for HBoV.

## 4 DISCUSSION

Since its discovery in 2005, it became more and more evident that the human bocaviruses are serious pathogens^4^, ^16^, ^17^, ^18^, ^19^ being involved in acute and chronic disease development. The testing of some HBoV research‐related hypothesis, however, is still hindered by the lack of an animal model and the restriction of cell culture models to some selected laboratories due to the need of highly specialized cell lines that are not freely available.^20^, ^21^ Moreover, it was shown independently by several labs that HBoV is able to persist in the infected host,^9^, ^10^, ^11^ which in turn makes it difficult to discriminate between an active contribution to a given clinical situation or a simple co‐existence or co‐detection due to the viral persistence. Thus, there is still an urgent need to retrospectively address the question in which clinical entities HBoV occurs in order to set up future prospective studies that may allow even these discriminations.

Under these prerequisites, our study shows that HBoV may play a role in the development of abortions, not at least as its two closest relatives, the canine parvovirus and the bovine parvovirus, as well as the human parvovirus B19 are responsible for even this clinical entity in their respective hosts. The fact that despite a positive PCR result no histological signs of the HBoV infection such as cytopathic effects were observed in histology fits to the fact that the virus induces only minor or moderate effects in infected cell culture. The stroma fibrosis observed in one case also is in agreement with earlier observations that HBoV is able to induce fibrosis in infected organs.^22^

However, it has to be stated that the present study cannot demonstrate if the miscarriage was caused by HBoV or was just accompanied by the virus, although the presented data increase the likelihood of a causal involvement. Instead, our observations contradict the findings by Enders and colleagues^13^ and Riipinen and coworkers.^12^ Enders et al tested amniotic liquid for HBoV which may have been less positive as HBoV may not be shed into this liquid but appears to be more tissue bound. Viral shedding is dependent on the three‐dimensional structure of the infected tissue^23^ and thus may not have been supported by fetal or placental tissue. In contrast, Riipinen et al, also tested FFPE samples as used in our study, but did not find HBoV DNA in their samples; most likely, the DNA extraction used in our laboratory, that is, the Maxwell DNA FFPE methods (Promega), which is the current Gold standard for FFPE DNA extraction, enable more sensitive analyses and thus may have caused the discrepancy.

Unfortunately, in this context we were not able to test the patients for their parvovirus B19 status or other pathogens except Zikavirus,^15^ as due to the retrospective nature of the study no corresponding sera were available. It thus cannot be excluded that also other pathogens may have played a role in the investigated cohort or may even have interacted with HBoV, thus this aspect should be addressed in future clinical studies. These future studies should also in depth evaluate to which extent HBoV may be also present in abortion tissues with other know causes of death as Chlamydia, herpes viruses or non‐infectious causes. Nevertheless, the fact that HBoV was not detected in the control cohort increases the likelihood of a causal involvement of HBoV in miscarriage. In summary, the fact that 25% of abortions had a positive result for HBoV DNA could lead to the careful conclusion that HBoV may be serious pathogen also during pregnancy. Thus, unless an animal model will be available, more prospective in‐depth studies are required to elucidate this pathogen's role in miscarriages.

## CONFLICT OF INTEREST STATEMENT

None of the authors declares and conflict of interest related to this study.

## ETHICAL STATEMENT

All procedures were performed in agreement with the declaration of Helsinki and in agreement with a vote of the Ethics Committee of the Private University of Witten/Herdecke (vote no. 15/2017). Due to the retrospective character of the study and the anonymization of the samples no written informed consent was required.
